# Risk factors for early postoperative cognitive dysfunction after non-coronary bypass surgery in Chinese population

**DOI:** 10.1186/1749-8090-8-204

**Published:** 2013-11-01

**Authors:** Tao Xu, Lulong Bo, Jiafeng Wang, Zhenzhen Zhao, Zhiyun Xu, Xiaoming Deng, Wenzhong Zhu

**Affiliations:** 1 Department of Anesthesiology and Intensive Care Medicine, Changhai hospital, Second Military Medical University, Shanghai, China; 2 Department of Cardiothoracic surgery, Changhai hospital, Second Military Medical University, Shanghai, China

**Keywords:** Postoperative cognitive dysfunction, Cardiovascular surgery, Risk factor, Sevoflurane

## Abstract

**Background:**

The present study was performed to investigate the incidence of early postoperative cognitive dysfunction (POCD) after non-coronary bypass surgery and the potential risk factors in Chinese population.

**Methods:**

We performed a prospective study in a teaching tertiary hospital from May 2012 to August 2012. One hundred and seventy-six adult patients undergoing non-coronary bypass surgery were recruited. Mini-Mental State Examination (MMSE) score was evaluated before and 3 to 5 days after surgery. Patients with a MMSE score reduction of 2 was diagnosed with POCD.

**Results:**

The general incidence of POCD was 33.0%, with no significant difference between the types of surgeries. In the univariate analysis, POCD associated factors included age, duration of surgery, anesthesia, cardiopulmonary bypass (CPB), cross-clamp and rewarming, and sevoflurane concentration. However, only age, cross-clamp duration and sevoflurane concentration were demonstrated to be independent risk factors for POCD.

**Conclusion:**

Incidence of early POCD after non-coronary bypass surgery was relatively high in Chinese population. Advanced age, longer aortic cross-clamp duration and lower sevoflurane concentration was associated with a higher incidence of POCD.

## Background

Postoperative cognitive dysfunction (POCD) is a common neurologic complication after cardiovascular surgery with an incidence varying between 33% and 83% [1-3]. A considerable proportion of patients with POCD (7 ~ 69%) do not recover in 3 months after surgery [4,5]. Although the cognitive changes was not manifested clinically in some patients, it may lead to prolonged hospital stay, elevated medical cost, increased morbidity, declined life quality and readmission to hospital [6].

Although POCD has been concerned by cardiovascular surgeons and anesthesiologists since 1950s, the exact etiology of POCD remains unclear. It was found that brain swelling develops in 4 days after cardiopulmonary bypass (CPB) and tends to disappear at the end of first postoperative week [7-9]. Beside the structure changes, both electroencephalograph (EEG) and P300 latency of evoked cognitive potential show functional change after CPB too [10,11]. Unfortunately, all of brain changes that previously observed did not clarify the pathogenesis of POCD completely. What’s more, those of changes did not parallel with cognitive status. It was reported that intraoperative hypotension and multiple microembolization are major reasons for POCD development [12,13]. But in clinical settings, previous research revealed that multiple risk factors were associated with cognitive deterioration after cardiac surgery, such as left ventricular function, presence of diabetes, duration of anesthesia, a second operation, age and a history of neurologic disease and so on [14-17]. These issues were thought to be susceptible to intraoperative hypotension and microembolization which leaded to POCD.

However, most of the data in previous studies were derived from coronary artery bypass graft (CABG) surgeries. As most of cardiovascular surgeries are performed on pump, such as congenital heart disease and rheumatic valve disease, the patients are also confronted with intraoperative hypotension and multiple microembolization. Therefore, patients undergoing cardiovascular surgeries other than CABG experienced similar pathophyisologic risks responsible for POCD. However, there were few reports of POCD after non-coronary bypass surgery. On the other hand, there may be differences in anatomy of brain vessels between the Chinese and the Caucasian [18,19]. It is unclear whether there is any differences in the incidence of POCD in Chinese population.

The objective of this study was to evaluate the general incidence of early POCD after non-CABG cardiovascular surgery and discuss the clinical factors associated with early POCD in first postoperative week which influence the patients without a history of neurologic disease.

## Methods

The study was approved by the special committee on ethics of biomedicine research of our university and written informed consent was obtained from all patients.

A total of 182 patients undergoing non-coronary bypass surgery were included in this study from May 2012 to August 2012. Inclusion criteria were defined as follows: (1) scheduled for a cardiovascular surgery, (2) at least 18 years old and (3) be able to speak and read Chinese. Exclusion criteria were as follows: (1) Mini-Mental State Examination (MMSE) score < 24 before surgery, (2) emergency cases, (3) a history of drug or alcohol abuse, (4) a history of psychiatric disorder, (5) history of a neurologic disease (including stroke history),(6) previously undergone neuropsychological testing, (7) had any severe visual or auditory disorders, (8) had Parkinson’s disease, (9) were unwilling to comply with the protocol or procedures.

Patients were interviewed in their hospital unit one day before the surgery. Test was carried out in quiet rooms and the patient and investigator were present. If the patient could not be tested in the test room, a quiet setting on the ward would be found for the test. All neuropsychological evaluations were conducted by trained research assistants. The training of the research assistants took an average of 72 hours, and supervision was performed throughout the project. The evaluation took an average of 50 minutes to complete and the second time was performed in the hospital unit during the period of 3 day to 5 day after surgery. The MMSE consists of cognitive functions of orientation, attention, calculation, memory and language. A control group was established involving 16 patients with valvular diseases who did not receive surgical treatment for the z value calculation. The formula for z score calculation is as follows: z score = ([change score] – [mean change score_control_])/(standard deviation change score_control_). POCD was defined as a MMSE deterioration of 1 z score [15,17].

Patients did not receive premedication. In the operating room, standard monitoring was used, including a 5-lead electrocardiogram, digital pulse oximeter, capnography, radial arterial catheter. Anesthesia was induced with midazolam (0.07 ~ 0.1 mg/kg), sulfentanyl (0.7 ~ 1.0 μg/kg), rocuronium (0.6 ~ 0.9 mg/kg) and etomidate (0.3 ~ 0.4 mg/kg). Intubation was done after induction administration over 1 min. Ventilation support was given immediately after intubation with O_2_ in air (60%). Anesthesia was maintained with sevoflurane and intravenous infusion of sulfentanyl (0.8 μg/kg/h) and cisatracurium (0.2 mg/kg/h), and supplemental bolus of intravenous sulfentanyl, midazolam, and rocuronium administered before skin incision, sternotomy, aortic cannulation, and sternal retraction.

Surgery procedures were classified as on-pump valve replacement, valvuloplasty, Bentall procedures, and repair of ventricular septal defect and/or atrial septal defect. Except some of congenital heart disease, a middle line sternotomy incision was made in all cases. Anterio-lateral thoracic incision on right side was made in some of congenital heart disease.

Heparin was given in 400 unit/kg dosage before ascending aortic intubation central line for systemic heparinization and additional dosage was given (80 u/kg) per 30 min to maintain activated coagulation time > 500 s. Systemic hypothermia was initiated after CPB started and target temperature was achieved according to the surgery classification. CPB flows were maintained between 2.2 ~ 2.5 L/min/m^2^. Mean blood pressure was maintained between 45 ~ 75 mmHg during the bypass. Myocardial protection during CPB consisted of an antegrade cold blood cardioplegia administered at regular intervals (20 minutes), local hypothermia, and systemic hypothermia. After aortic clamp removed, inotropic agents were used according to cardiac performances and hemodynamic status. Weaning from CPB was conducted when cardiac main procedure finished and hemodynamic status was stable and agreement with surgeon achieved.

All statistical analysis was performed in SPSS software. Continuous measurement data was shown as mean ± SD and compared using student’s t-test. Semiquantitative data and numerous data were compared using Chi-square test. Multivariate data was analyzed by logistic regression analysis using a stepwise backward method. *p* < 0.05 was considered as statistically significant.

## Results

Among the 182 recruited patients, 4 were excluded because of re-operation for bleeding and 2 were excluded because of refusal to neuropsychological evaluation. The final number of patients included in the analysis was 176. The general demographic data of patients were shown in Table 1, including age, gender, body mass index, education background, surgery type, previous history of hypertension, ASA classification, NYHA cardiac function class and ejection fraction. Anesthesia and surgery related information were also listed, such as position, surgery and anesthesia duration, CPB and cross-clamp duration, rewarming duration, dose of anesthetics (Table 2). According to MMSE results, there were 58 patients meeting the diagnosis of POCD (33.0%).

**Table 1:** Demographics of patients

**Items**	**Value**
Age (years)	41.7 ± 18.7
Gender (M/F)	94/82
BMI	21.0 ± 3.9
Education background	
<3 years (%)	14 (8.0)
3-6 years (%)	50 (28.4)
7-9 years (%)	64 (36.4)
9-12 years (%)	32 (18.2)
>12 years (%)	16 (9.1)
Surgical type	
Congenital disease(%)	52 (29.5)
Valvular disease (%)	86 (48.9)
Aorta disease (%)	34 (19.3)
Tumor (%)	4 (2.3)
Hypertension (%)	26 (14.8)
ASA (II/III/IV)	102/56/18
NYHA (I/II/III/IV)	21/97/50/8
EF (%)	61.4 ± 8.3

NOTE. Data were expressed as number (%) or mean ± standard deviation. M, male; F, female; BMI, body mass index; ASA, American Society of Anesthesiologists score; NYHA, New York Heart Association cardiac functional classification; EF, ejection fraction.

**Table 2:** Surgical and anesthetic data

**Items**	**Value**
Head-down position (%)	142 (80.7)
Surgical duration (h)	3.3 ± 1.0
Anesthesia duration (h)	4.1 ± 1.2
CPB duration (min)	89.3 ± 33.1
CC duration (min)	50.6 ± 23.1
Rewarming duration (min)	33.6 ± 1.6
Midazolam dose (mg)	10.6 ± 2.9
Etomidate dose (mg)	25.4 ± 9.9
Sufentanil dose (μg)	262.8 ± 107.1
Sevoflurane concentration (%)	2.0 ± 1.1

NOTE. CPB, cardiopulmonary bypss; CC, cross-clamp.

### Univariate analysis

Univariate analysis was performed to find any potential risk factor of POCD (Table 3). Patients with POCD were significantly older than non-POCD patients (p = 0.040). More patients in POCD group belonged to ASA classification 3 and 4. Duration of surgery, anesthesia, CPB and cross-clamp were closely related to each other and all of them were correlated with POCD (p < 0.01). Non-POCD patients had a higher concentration of sufentanil and longer rewarming duration (p < 0.01), the time interval from the start to finish of body temperature rewarming. Surgery type was less likely to impact the incidence of POCD (p = 0.051).

**Table 3:** Univariate analysis of early POCD after non-CABG cardiac surgery

**Items**	**POCD**	**Non-POCD**	***P *** **value**
Age	45.9 ± 17.9	39.6 ± 19.8	0.040
Gender (M/F)	32/26	62/56	0.742
BMI	20.5 ± 3.6	21.2 ± 3.9	0.239
Education background			0.122
<3 years (%)	3	11	
3-6 years (%)	22	28	
7-9 years (%)	22	42	
9-12 years (%)	10	22	
>12 years (%)	2	14	
Surgical type			0.051
Congenital disease	10	42	
Valvular disease (%)	32	54	
Aorta disease (%)	14	20	
Tumor (%)	2	2	
Hypertension (%)	10	16	0.518
ASA (II/III/IV)	24/24/10	78/32/8	0.005
NYHA (I/II/III/IV)	10/33/18/2	11/66/32/6	0.309
EF (%)	61	63	0.688
Hypertension (%)	62.8 ± 8.5	60.8 ± 8.1	0.173
Head-down position (%)	15.4	10.9	0.419
Surgical duration (h)	3.7 ± 1.4	3.1 ± 0.8	0.003
Anesthesia duration (h)	4.6 ± 1.5	3.8 ± 0.8	0.001
CPB duration (min)	105.2 ± 37.8	82.1 ± 28.0	<0.001
CC duration (min)	61.3 ± 26.0	45.8 ± 19.9	<0.001
Rewarming duration (min)	32.8 ± 2.2	34.0 ± 1.1	<0.001
Midazolam dose(mg)	10.3 ± 3.5	10.7 ± 2.6	0.502
Etomidate dose (mg)	27.2 ± 11.1	24.4 ± 9.1	0.108
Sufentanil dose (μg)	282.2 ± 118.8	252.8 ± 99.8	0.122

NOTE. Date are expressed as number (%) or mean ± standard deviation. M, male; F, female; BMI, body mass index; ASA, American Society of Anesthesiologists score; NYHA, New York Heart Association cardiac functional classification; EF, ejection fraction. CPB, cardiopulmonary bypass; CC, cross-clamp. Variables with a p < 0.01 was included in the multivariate analysis.

### Multivariate analysis

The factors with a p value lower than 0.1 in the univariate analysis were screened for multivariate analysis, including age, surgery type, ASA classification, surgery duration, anesthesia duration, CPB duration, cross-clamp duration, rewarming duration and sevoflurane concentration.

Among these factors, continuous measurement data were converted to discrete data and then all the factors were admitted into logistic regression analysis. Finally, the independent risk factors for POCD were age (p = 0.043), cross-clamp duration (p = 0.028) and sevoflurane concentration (p < 0.001) (Table 4).

**Table 4:** Multivariate analysis of early POCD after non-CABG cardiac surgery

**Items**	**Coefficient**	***P *** **value**	**OR (95% CI)**
Age	0.29	0.043	1.34 (1.01-1.78)
Anesthesia duration	0.44	0.053	1.55 (1.00-2.41)
CC duration	0.77	0.028	2.167 (1.09-4.32)
Sevoflurane concentration	-0.68	0.000	0.505 (0.34-0.74)

NOTE. CC, cross-clamp.

## Discussion

POCD is a common complication of cardiovascular surgery affecting patients’ outcomes. Early POCD will complicate the medical disposal, and prolong the hospitalization. The exact etiology of POCD remains unclear. The most accepted risk factors are periopertive hypoperfusion and microembolism. As a significant unphyisological circulation, CPB plays an important role to introduce the both predisposing factors to POCD [20]. Previous study revealed that the use of CPB is an independent factor associating with POCD after CABG [5,7,9]. There are few researches on general incidence of POCD after non-CABG but on pump cardiovascular surgery [10]. However, most of cardiovascular surgeries are performed on pump. As the patients went on the same predisposing factor, CPB, whether they confront with same risk to the POCD remains unknown. Moreover, although the incidence of cognitive dysfunction after CABG in Chinese population was reported by Liu et al. [21], there is no report on general incidence of POCD in Chinese population undergoing non-CABG cardiovascular surgery. So the study was focused on incidence of POCD after cardiovascular surgery involving non-CABG cardiac surgery, as well as the perioperative risk factors in Chinese population.

In the present study, the general incidence of early POCD was 33.0% within 7 days after surgery and the difference of POCD incidences after different surgery was not statistically significant. Although incidence of POCD after aortic and tumor surgery seemed higher than that of other types of surgery, the phenomenon might be due to longer surgery and CPB duration. Head-down position was always applied during aorta opening to prevent cerebral air emboli in many centers in China. However, our results showed no correlation between head-down position and incidence of POCD, which is in accordance with a previous study which revealed that cerebral microemboli were not significant related to the occurrence of POCD in Chinese population [21].

Cross-clamp duration was recognized to be an independent risk factor for POCD after non-CABG cardiac surgery, but not duration of CPB, anesthesia or surgery. Circulation status during cross-clamp period was special because of completely artificial circulation. Two causes of the perfusion status might contribute to POCD during cross-clamp period. First, cross-clamp might lead to cerebral hypoperfusion during cross-clamp period, which was deemed as an important factor of POCD in non-cardiac surgery. Ogasawara et al. reported that cognitive dysfunction without neurologic deficits were associated with perioperative hypoperfusion in carotid endarterectomy [22]. Unfortunately, the optimal perfusion pressure during CPB for cerebral perfusion remains unclear. Some studies suggested that higher perfusion pressure might be benefit for neurologic deficit patients, but some other studies demonstrated that perfusion pressures too high or too low were both harmful to cognitive function [23]. Therefore, the potentially inappropriate perfusion pressure might be the reason that longer cross-clamp duration lead to POCD. Second, non-pulsate CPB, the only circulation during the aortic cross-clamp period, might result in poorer tissue perfusion than physiological pulsate flow, which was the other reason of POCD during cross-clamp period [24]. In general, ischemia or hypoxia induced by hypoperfusion or non-pulsate flow was a key contributor to POCD.

The etiology of POCD by hypoperfusion is still unknown. But it can be inferred that hypoperfusion will make energy deprived in perfusion area. Many proapoptotic genes were activated by downregulation of the ATP level in mitochondrion. Typically, anti-apoptosis members of Bcl-2 protein family became activated and act on the mitochrondial to release cytochrome C, then apoptosis was induced. More severe energy exhaustion would make the cells directly to necrosis [25]. On the other hand, restoration of perfusion will induce humoral and cellular activation, leading to a systemic inflammatory response, which may contribute to incidence of POCD [26].

Interestingly, sevoflurane was found to be an independent factor for POCD in the present research. There were many reports about sevoflurane preconditioning for cardiac protection in cardiac surgery. Sevoflurane and other volatile anesthetic have been showed to reduce myocardial infarction and, perioperative death and postoperative inotrope drug dosage. But effect of sevoflurane on neural system was still controversial. Animal study revealed that sevoflurane impaired memory consolidation in rats [27]. However, it was also reported that postconditioning of sevoflurane protected against focal cerebral ischemia in rats, as demonstrated that sevoflurane preconditioning attenuated neurological deficit score, cerebral infarct volume and brain edema [28]. Several clinical trials favored inhalational anesthetics which showed a protective effect against cognitive dysfunction. Delphin et al. [29] revealed that patients undergoing fast-track anesthesia with sevoflurane as prime maintenance anesthetic for OPCAB were extubated earlier and allowing assessment of cognitive and neurologic function earlier than those receiving isoflurane. A recently published randomized controlled study showed that desflurane leaded to a reduced incidence of early POCD before discharge from hospital among patients underwent coronary artery bypass surgery [30]. Therefore, it was not strange that our present study showed that higher concentration of sevoflurane concentration was associated with a lower incidence of POCD after non-CABG surgery. But whether sevoflurane was protective against POCD should be further confirmed by well-designed randomized controlled trials.

## Conclusion

In summary, the incidence of POCD is relatively high after non-CABG cardiovascular surgery in Chinese population. Advanced age, longer aortic cross-clamp duration are the potential risk factors, while higher sevoflurane concentration is a potential protective factor.

## Abbreviations

POCD: Postoperative cognitive dysfunction; CABG: Coronary-artery-bypass-graft; MMSE: Mini-Mental State Examination; CPB: Cardiopulmonary bypass; EEG: Electroencephalograph; PAC: Pulmonary artery catheter.

## Competing interests

All authors declare that they have no competing interests.

## Authors’ contributions

WZ was responsible for all data of the study and modified the manuscript. TX and LB performed the trial and collected all data. TX and LB wrote the draft. JW and ZZ did the anesthesia during the cases. ZX and XD helped to design the study. All authors read and approved the final manuscript.
